# Prevalence of smoking among male secondary school students in Arar City, Saudi Arabia

**DOI:** 10.11604/pamj.2019.32.156.18558

**Published:** 2019-04-08

**Authors:** Fahed Hlilan Albangy, Amal Elwan Mohamed, Sabry Mohamed Hammad

**Affiliations:** 1 Saudi Board of Family Medicine, Northern Borders Region, KSA, Saudi Arabia; 2 Faculty of Medicine, Zagazig University, Cairo, Egypt; 3 Faculty of Medicine Mansoura Egypt, Consultant of Public Health Northern Borders General Health Affairs, KSA, Saudi Arabia

**Keywords:** Tobacco, smoking, adolescents

## Abstract

**Introduction:**

Tobacco is one of the leading preventable cause of death worldwide. Tobacco consumption among teenagers is a major public health problem, especially in developing countries. Younger smokers are more liable to smoking complications. The objectives were to evaluate the prevalence of smoking among male secondary school students in the Northern Borders region, KSA.

**Methods:**

A cross-sectional study was conducted in Arar city. Four schools were chosen randomly from 21 secondary schools. A total of 240 students responded to the pre-designed questionnaire. The questionnaire included questions on demographic factors, smoking behavior and knowledge about smoking hazards.

**Results:**

Prevalence of current smoking among male secondary school students is 40.8%. Cigarette smoking was the most common type (67.3%) followed by Shish smoking (22.4%). Few students (2.1%) reported other forms of smoking, example (Hashish). Of the studied group, 39.8% smoke on a daily basis with 29.6% of them smoke more than five cigarettes per day.

**Conclusion:**

Smoking is a prevalent habit among teenagers. Special concern should be directed to smoking cessation campaigns with behavioral, legal and economic interventions.

## Introduction

According to the world health organization (WHO) reports, tobacco use is the leading cause of preventable death. The Smoking epidemic is one of the most challenging public health threats the world has ever faced, causing more than 7 million deaths a year. More than 6 million of those deaths are the result of direct tobacco use while around 890 000 are the outcome of passive smoking. By the year 2030, tobacco is likely to be the world's leading cause of death and disability killing more than 8 million people annually especially in developing countries [1]. WHO recommends more health programs for adolescents because they are in the critical period in their life as during this period they usually acquire experiences, knowledge and skills which have significant effect their adulthood life [2]. There is strong relationship between smoking and adolescents as WHO reported that among adolescents, about one in five smokers worldwide. Also, it stated that around half of those who start smoking at a young age go on to smoke for fifteen to twenty years [3]. Also, it had been documented that smoking is a gateway to other illicit drug addiction. Adolescents who smoke are three times more likely to use alcohol, eight times more likely to use marijuana, and twenty-two times more likely to use cocaine [4]. Tobacco smoke contains a deadly mix of more than seven thousand chemicals. Hundreds of them are toxic and about seventy can cause cancer [5]. Globally, tobacco is used in many different ways as it can be smoked like cigarettes, cigars, pipes and water pipes or smokeless tobacco in the form of chewing tobacco and snuff [6].

Smoking affects both smokers and people around smokers (passive smokers). Nonsmokers exposed to passive smoke in everyday life exhibit an increased risk of both fatal and nonfatal cardiac events [7]. The most important causes of smoking-related mortality are lung cancer, atherosclerotic cardiovascular disease (CVD) and chronic obstructive pulmonary disease (COPD) [8, 9]. The most critical factors that contribute to increasing the risk of smoking among adolescents include peer pressure through siblings' and/or friends' smoking, tobacco industry advertising and easy access to tobacco products and their low prices [7]. In Saudi Arabia, in Jeddah 2013 the prevalence of smoking was 37% [10] in hail, a survey study of cigarette smoking found that 19.5% of adolescent students were current smokers [11]. Growing or manufacture of tobacco is not allowed in Saudi Arabia, despite that, approximately US$ 150 million (600 million SR) are spent annually on tobacco [12]. In Saudi Arabia, there is an urgent need for research evaluating tobacco control program and its effect on the prevalence of tobacco use. Smoking is highly prevalent in the Saudi population at different age groups, much higher in males than in females. The prevalence of current smoking among the Saudi population ranges from 2.4-52.9% with a median of 17.5% [13]. Tobacco is the single, most main preventable cause of premature death and the most important public health issue in the current time. There is an ongoing increase prevalence of smoking among adolescent worldwide and especially in Saudi Arabia. The study aims to enhance tobacco control program through identification of secondary school male students' smoking pattern and investigation of their knowledge regarding smoking hazards.

## Methods

This is a cross-sectional study conducted in Arar city. Arar is the capital of Northern Borders Province, Kingdom of Saudi Arabia. It has a population of 166.512 (2010 census). In Arar, there are 21 secondary schools for normal students including 5780 students.

**Inclusion criteria**: male gender, adolescent age, resident in Arar city and regularly attending student.

**Exclusion criteria:** students who have a hearing limitation or limited intellectual capabilities. The sample size for this study has been calculated according to this formula [14] as follows:

$$n=\frac{\text{Z}2*\text{P}*\text{Q}}{\text{D}2}$$

n: calculated sample size, Z: the z-value for the selected level of confidence (1- a) = 1.96. P: an estimated prevalence of smoking among male secondary school students was assumed as 37% according to a similar Saudi study carried out in Jeddah (2013) [10]. Q: (1-0.37) = 63%, i.e, 0.63. D: The maximum acceptable error = 0.05. So, the calculated minimum sample size was 204. This sample was increased to 240 students to compensate for none or incomplete response of students. There were 21 secondary schools for boys in Arar city. Regular secondary school male students during the 2016-2017 academic year were 5,780 boys. Sample was chosen by multistage cluster sample: three schools were randomly selected by a simple random technique. Within each school, one class was selected representing each grade. Thus three classes were chosen from each school and a total of 12 classes were recruited. All students from each class completed the questionnaire. Data was collected using a self-administered questionnaire. It included three main sections. The first section includes the socio-demographic characteristics of the students (age, level, nationality, parental education and job). The second section inquires about the smoking behavior of the students. The third section concerns with knowledge of students about smoking hazardous effects and their attitude towards smoking cessation. The second and third sections were based on the Arabic version of the Global School-based Student Health Survey (GSHS, 2005) [15]. The questionnaire was validated and tested for reliability. The researcher distributed the self-administered questionnaire on students with the help of teachers during the break time. Care was taken not to disturb the students' school activities as much as possible. The researcher clarified any issue and the questionnaires were collected on the same day. This was done over approximately 3 months period from 1^st^ Feb till 30^th^ April 2018. A pilot study was conducted on 20 students of one secondary school other than the selected schools. The pilot study helped to test the students' understanding of the questionnaires and determine the time needed to answer the questionnaire. Data were analyzed using Statistical Package for Social Sciences (SPSS) software version 20.0. Descriptive statistics (e.g., number, percentage) were used for qualitative data and analytic statistics using Chi-Square tests (χ^2^) to test for the association. P-value equal or less than 0.05 was considered statistically significant. Written permission from Joint Program of Family Medicine, Arar was obtained before conduction of the research. Ethical approval was taken from the local ethical committee in Northern Borders Region NO 39/1. Written permission from related authority from the ministry of education was obtained. Feedback of the result and recommendation was submitted to concern authority.

## Results

Table 1 showed demographic data of studied group about smoking habit there was no statistically significant difference between smokers and non-smokers as regard parental occupation and education. Seventy-One percent of non-Saudi were smokers compared to 39.9% of Saudi students were smokers and the difference was statistical significance (p = 0.03). Table 2 showed that the prevalence of current smoking among the studied group was 40.08%. Sixty-Seven percent were cigarettes smoker, 22.4 smoked shishas while 2.04 reported smoking of others (hashish). Twenty-nine percent 29% smoked more than five cigarettes per day. Nearly 40% of them smoked on a daily basis. Table 3 revealed that no statistically significant difference between smokers and non-smokers regarding information received on smoking hazards. Having one family or friend member smoker washe study and confirming confidentially of the data.Feedback of the result and recommendation was submitted to concern authority.

**Table 1:** Demographic data of the studied group in relation to smoking habit (total=240)

Item		Total	Smoker 98 N(%)	Non- smoker 142 N(%)	P
**Age**					0.3
15		3	0	3(100)	
16		74	28(37.8)	46(62.2)	
17		81	27(33.3)	54(66.7)	
More than18		82	43(52.4)	39(47.6)	
**Nationality**					
Saudi	233		93(39.9)	140(60.1)	0.03
Non-saudi	7		5(71.4)	2(28.6)	
Grade					0.8
1^st^	95		37(38.9)	58(61.1)	
2^nd^	86		35(40.7)	51(59.3)	
3^rd^	59		26(44.1)	33(55.9)	
**Father Occupation**					0.5
No work	11		4(36.4)	7(63.6)	
Employee	35		18(51.4)	17(48.6)	
Skilled	37		15(40.5)	22(59.5)	
Military	54		20(37.1)	34(62.9)	
Businessman	8		5(62.5)	3(37.5)	
Retired	82		31(37.8)	51(62.2)	
Died	13		5(38.5)	8(61.5)	
**Working Mother**					
Yes	56		23(41.1)	33(58.9)	0.9
No	184		75(40.7)	109(59.3)	
**Father education**					0.5
Illiterate	25		8(32.0)	17(68.0)	
Read	45		16(35.5)	29(64.5)	
Middle & secondary	100		46(46.0)	54(54.0)	
Postgraduate	70		28(40.0)	42(60.0)	
**Mother education**					0.5
Illiterate	60		21(35.0)	39(65.0)	
Read	48		21(43.8)	27(56.2)	
Middle & secondary	76		35(46.1)	41(53.9)	
Postgraduate	56		21(37.5)	35(62.5)	

**Table 2:** Smoking pattern among studied group (total=240)

Item	N	%
Prevalence of current smoking	98	40.8
Family member is smoker	144	60
Friends are smokers	169	70.4
**Smoking types n=98**		
Cigarette	66	67.3
Shisha	22	22.4
Hand warped cigarette	4	4.08
Electronic	4	4.08
Other	2	2.05
**How many cigarettes you smoked last month? n=98**		
None	33	33.6
Less than 1	5	5.10
I cigarette	14	14.3
2-5 cigarettes	17	17.4
>5 cigarettes	29	29.6
**How many days you are smoke in the last 30 days? n=98**		
No	26	26.5
1 or 2 days	6	6.1
3-5 days	7	7.13
6-9 days	3	3.07
>9 days	17	17.4
Every day	39	39.8
**Attempted to quit? n=98**		
Yes	67	68.4
No	31	31.6

**Table 3:** Some risk factors related to smoking

Item	Total	Smoker (98) N(%)	Non Smoker (142) N(%)	P(OR)
**Received information on smoking**				
yes	184	72(73.4)	112(78.8)	0.4
No	56	26(26.6)	30(21.2)	0.7(0.4-1.3)
**Knows hazards of smoking**				
Yes	209	89(90.8)	120(84.5)	0.1
No	29	9(9.2)	20(15.5)	1.7(0.7-4.2)
**Ever Tried to smoke**				
Yes	126	98(100)	28(19.7)	0.00**
No	114	0(0)	114(80.3)	75.7(28.1-203)
**Family member is smoker**				
Yes	144	75(76.5)	69(48.5)	0.000**
No	96	23(23.5)	73(51.5)	3.4(1.9-6.1)
**Friends are Smokers**				
Yes	169	87(88.7)	82(57.7)	0.000**
No	71	11(11.3)	60(42.3)	5.7(2.8-11.7)

** Highly significant

## Discussion

Smoking among teenagers is a problem of rising concern. This study aims to assess prevalence of smoking and describe its pattern among male secondary school students Arar city. This is a cross-sectional study conducted on 240 students. Of the studied group, (40.8%) were current smokers. Half of the students tried to smoke. Cigarette smoking was the most common type (67.3%) followed by Shish smoking (22.4%). Few students (2.1%) reported other forms of smoking, e.g. (Hashish). It was striking that (39.8%) smoked daily last month with 29.6% smoked > 5 cigarettes per day. There was no statistical difference between smokers and nonsmokers regarding socio-demographic factors. Having a family member or a friend who smoke were the only significant factors. The prevalence of smoking was high compared to other regions in KSA [10, 11]. In Riyadh, One in five students reported having ever smoked cigarettes. Having family members who smoke was frequent among the studied group. Students with family members who smoked were significantly more likely to use tobacco themselves [16]. Another recent study In Riyadh also found that the prevalence of smoking was 24.3% among adolescents. Several personal and social factors were identified as important determinants for smoking as having a family member who smokes and smoker friends [17]. In Qassim, Saudi Arabia, Al Nohair found that Out of these 680, 161 students (23.6%) were smokers. Students who had smoking friends were 2.3 times more likely to smoke (OR=2.35, 95% CI=1.49-3.70, P < 0.001) [18]. There is variability in the reported prevalence of smoking in these studies. This may be explained that the prevalence of smoking among adolescents might have been underestimated in some settings. Some investigated adolescent boys were ashamed, to tell the truth about their smoking habit because of cultural and religious factors. This indicates that smoking is an increasing problem among adolescents. The effect of family and peers was evident. The current study showed that adolescents having a family member who smoke were three times more likely to smoke. Also, those who had smoking friends were 5 times more likely to smoke.

This is in agreement with a study in Jeddah (Saudi Arabia), rate of current smoking among the students was (37%). Majority of current smokers (83.7%) had started smoking at the age of 14 years or less. The most frequently reported reason for smoking was the influence of family (65.9%) and friends (42.5%). Also, almost two-thirds of the students (66.3%) searched for information on the risks of smoking and only (45.3%) knew about the adverse effects of passive smoking on others [10]. Regarding attemps to quit smoking, the present study demonstrated that 68.4% of smokers attempted to quit but failed and this is a considerable percentage. In Jaddah study, two-third of the students who smoked (63.2%) wanted to quit smoking whereas 60.9% had tried to quit [10]. The reported prevalence in the current study is much higher than other regions in Ksa. For example in a study in Jazan, Gaffar *et al*. found that the prevalence of smoking was 17.3% and the current smoking prevalence was 10.7%. The most important risk factors for smoking had friends who used khat (OR = 3.23) and having friends who used tobacco (OR = 2.88) [19]. This is in agreement with the present study. In Riyadh, Al Nohair stated that current smokers' prevalence rate was 28.6% [20]. In Al-Qassim, Al-Damegh and his research team carried out a cross-sectional study to investigate smoking behavior among male secondary school students and evaluate their knowledge and attitudes towards smoking. The prevalence of current smoking was 29.8%. Among them, 83.7% started smoking at the age of 15 years or less. The most common reasons given for cigarette smoking behavior were the influence of friends (63.5%) and brother's smoking habit (24.8%). Most of the students knew that smoking is harmful to their health (89.3%) and others (73.9%) [21].

In Alkharj, Al-Yousaf and Karim found that current smoking rate was 20%, whereas the ex-smoker rate was 16%. Almost two-thirds (63%) of current smokers smoked less than ten cigarettes per day. The influence of friends (58%) and the presence of smoking in the family (32%) were the two most important factors influencing the rate of smoking in current smokers. Majority of the students (95%) knew that smoking is harmful and 60% know that smoking is harmful to others. Sixty-one percent of current smokers, have tried to quit smoking but failed [22]. The reported rate of smoking is higher than what is reported internationally, In Eastern Ethiopia, The prevalence of cigarette smoking was 12.2%. The main predictors were sex (OR 4.32), age (OR 1.20) and having friends who smoke (OR 8.14). Having family members who smoke cigarettes was not significantly associated with smoking in this age group (OR 1.25) [23]. In Zimbabwe, Prevalence of ever-smoking was 28.8%; among male and female students was 37.8% and 18.5%, respectively, p < 0.001. Smoking was significantly associated with alcohol use (OR 5.7), having friends that smoke (OR 2.8), marijuana use (OR 8.1) [24]. In Malaysia, The prevalence of smoking was 32.8% [25]. In Sudan, The prevalence of current smoking was 13.6%. Smoking among parents, other family members and close friends was the main factor for initiation of smoking [26]. In Botswana, Ten percent of students were current tobacco smokers with 29% reporting having tried smoking. The strongest predictors of smoking overall were self-image and acceptance by peers. Intention to smoke or to continue to smoke were also independently associated with smoking [27]. In Nigeria, The lifetime smoking prevalence was 26.4%, and current smoking prevalence was 17.1%. Most (82%) of the students had seen warnings against smoking. Most of them were aware of possible its complications. Smoker students were introduced to smoking mainly by their friends (67.4%) and the television (13.4%). Smoking habit was influenced by parents' educational status, having friends who smoke and living with a smoker [28]. There was no statistical difference between smoker and non-smokers regarding receiving information or knowledge of smoking hazards. It is obvious that knowledge alone doesn't grantee behavior. Modeling and targeted behavioral and legal interventions are needed. Role of media, peers and family cannot be ignored.

## Conclusion

Smoking is a growing problem among adolescents. School-based smoking cessation programs are necessary with the involvement of families. Further research is needed on smoking behavior to evaluate smoking control activities and to help policy-makers in their decision making.

### What is known about this topic

- Smoking is the main preventable risk factor for many diseases. This risky behaviour is common among adolescents.

### What this study adds

- Smoking is aprevalent habit among male secondry school students in Arar city, KSA despite of adequate knowledge of its hazards. This requires directed strategies to help them in quitting.

## Competing interests

The authors declare no competing interests.
